# Somatic mutation and expression of BAP1 in hepatocellular carcinoma: an indicator for ferroptosis and immune checkpoint inhibitor therapies

**DOI:** 10.7150/jca.65574

**Published:** 2022-01-01

**Authors:** Yu-Chuan Yan, Guang-Xiao Meng, Zi-Niu Ding, Yan-Feng Liu, Zhi-Qiang Chen, Lun-Jie Yan, Ya-Fei yang, Hui Liu, Chun-Cheng Yang, Zhao-Ru Dong, Jian-Guo Hong, Tao Li

**Affiliations:** 1 Department of general surgery, Qilu Hospital, Shandong University, Jinan 250012, P.R. China.; 2 Department of hepatobiliary surgery, The second Hospital of Shandong University, Jinan 250012, P.R. China.

**Keywords:** hepatocellular carcinoma, BAP1, ferroptosis, mutation, immune checkpoint inhibitors

## Abstract

BRCA1-Associated Protein 1 (BAP1) is a deubiquitylase that is found associated with multiprotein complexes that regulate key cellular pathways, and subsequent researches have revealed that BAP1 acts independently as a tumor suppressor. Somatic BAP1 mutations occur in various malignancies, but malignancies arising from mutation of tumor suppressors have unexplained tissue proclivity. Whether somatic mutation or expression alteration of BAP1 in hepatocellular carcinoma (HCC) influence carcinogenesis or immunogenicity is still unknown. In this study, we analyzed RNA expression, immune infiltration, survival and mutation data of HCC from The Cancer Genome Atlas databases. The association between BAP1 and clinicopathological features was further investigated by immunohistochemistry on tissue microarray. We found that the prognosis of patients with high BAP1 expression was significantly worse than that of patients with low BAP1 expression, and multivariate analyses revealed that BAP1 expression was an independent prognostic factor for poor prognosis. HCC with high BAP1 expression was associated with low ESTIMATE Score, recruitment of more tumor-infiltrating macrophage, and elevated levels of tumor mutation burden, microsatellite instability, neoantigen count, as well as programmed death-ligand1 in HCC. In addition, BAP1 mutated HCC showed reduced ability to promote ferroptosis and high BAP1 expression was correlated with ferroptosis. In conclusion, high BAP1 expression reflects immunosuppression and ferroptosis in HCC. BAP1 is a promising prognostic marker for survival of HCC and may act as a complementary indicator for patients to receive ferroptosis-promoting therapy or immunotherapy.

## Introduction

Hepatocellular carcinoma (HCC) is the sixth most commonly diagnosed cancer and the fourth leading cause of cancer death worldwide 1. Most HCCs develop in a chronically injured liver, and all of the major risk factors for HCC, which include hepatitis B (HBV) or C virus (HCV) infection, alcohol-induced liver damage, and nonalcoholic fatty liver disease, provide a chronic injury stimulus that leads to significant hepatic inflammation and fibrosis 2, 3. However, the extent to which this microenvironment promotes neoplastic transformation or influences selective pressures for genetic drivers of HCC remains unclear 2.

BRCA1-Associated Protein 1 (BAP1) is a nuclear deubiquitylase that is found associated with multiprotein complexes that regulate key cellular pathways, including the cell cycle, cellular differentiation, cell death, gluconeogenesis, gene expression, transcription and DNA repair 4. BAP1 was initially shown to localize to the nucleus where its primary interaction was binding to BRCA1 and enhancing its tumor suppressive activity 5, and subsequent researches have revealed that BAP1 acts independently as a tumor suppressor. Mutations in the BAP1 gene commonly result in cancers and recent findings indicate that germline BAP1 mutations cause a hereditary cancer syndrome with increased risk of mesothelioma and uveal melanoma. In addition, somatic BAP1 mutations are frequent in various human cancers, including uveal melanoma and mesothelioma, and have been noted in breast, lung, and renal cancers 6, but have not previously been appreciated as a significant somatic alteration in HCC.

The function of BAP1 is linked to its dual activity in the nucleus, where it is implicated in a variety of processes including DNA repair and transcription 7. BAP1 tumor suppressor activity has been attributed to its nuclear localization, where it helps to maintain genome integrity. However, malignancies arising from mutation of tumor suppressors have unexplained tissue proclivity 8. There were contradictory evidences supporting the tumor suppressor role of BAP1 in cancer. BAP1 activity could vary in different cells because the BAP1 core complex is likely to associate with different transcription factors and thus influence different pathways in a cell-type specific manner.

In this study, we aim to determine the somatic mutation and expression of BAP1 in HCC, and analyzed the associations of BAP1 expression with clinicopathological variables and clinical outcomes of HCC patients. In addition, we want to investigate the possible correlation and interaction of BAP1 with ferroptosis and immunogenicity in HCC, not only to identify BAP1 as a potential diagnostic and prognostic marker for HCC, but also identify it as an indicator for ferroptosis and immune checkpoint blockade therapies.

## Materials and Methods

### TCGA data sources Gene mutations in HCC

The Cancer Genome Atlas (TCGA) is a landmark cancer genomics program, molecularly characterized over 20,000 primary cancer and matched normal samples spanning 33 cancer types. In the TCGA database, there are multiple types of bioinformation, including transcriptional data, epigenetic data, genomic mutation profiles, and clinical data. We downloaded the genetic mutation data, transcriptome data, and clinical data from TCGA (http://portal.gdc.cancer.gov/). To identify the somatic mutations of HCC patients in the TCGA database, mutation data were downloaded and visualized using the “maftools” package in R software. Fold change was defined as 1.5 and p-value was set as 0.05.

### ESTIMATE score

ESTIMATE (Estimation of STromal and Immune cells in MAlignant Tumor tissues using Expression data) is an algorithm that takes advantage of the unique properties of the transcriptional profiles of cancer samples to infer tumor cellularity as well as the different infiltrating normal cells. ESTIMATE algorithm is based on single sample Gene Set Enrichment Analysis and generates stromal and immune scores to predict the level of infiltrating stromal and immune cells and these form the basis for the ESTIMATE score to infer tumor purity in tumor tissue 9.

ESTIMATE generates three scores: StromalScore (that captures the presence of stroma in tumor tissue); ImmuneScore (that represents the infiltration of immune cells in tumor tissue); EstimateScore (infers and negatively correlates with tumor purity). ESTIMATEScore was the sum of ImmuneScore and StromalScore denoting the comprehensive proportion of both components in TME 10.

### Correlation analysis of BAP1 expression and TMB/MSI, immune checkpoint genes

The dataset used comprised mRNA-seq data from TCGA LIHC tumors (https://tcga-data.nci.nih.gov/tcga/). We used Spearman's correlation analysis to describe the correlation between quantitative variables without a normal distribution. A p-value of less than 0.05 was considered statistically significant.

### Human Tissue microarray and immunohistochemistry

Tissue microarray (TMA, Shanghai Outdo Biotech Company) containing 297 pairs of tumors from HCC patients was used in this study. The inclusion criteria for these 297 patients were as follows: (i) all patients underwent curative resection; (ii) all patients were pathologically confirmed to have HCC; (iii) all patients did not receive tyrosine kinase inhibitors or immune checkpoint inhibitors therapy. Patients were excluded if: (i) they were pathologically confirmed to have combined HCC and cholangiocarcinoma; (ii) tumor recurred within one month after resection. Curative resection was defined as complete macroscopic removal of the tumor without exposure of tumor cells on the cut surface with macroscopic tumor clearance confirmed on a computed tomography (CT) scan or magnetic resonance imaging (MRI) study of the liver 1 month after hepatic resection 11. Tumor staging was determined according to the TNM classification system of the 8^th^ edition. The histological grade of tumor differentiation was assigned by the Edmondson grading system.

The TMA sections were used for immunochemistry staining. Monoclonal antibodies against human BAP1 (1:100), DPP4 (1:100) were purchased from DakoCytomation, Denmark. Immunohistochemistry was carried out using a two-step protocol (Novolink Polymer Detection System, Novocastra, Newcastle, UK) as previously described 12. Monoclonal antibodies against human PD-L1 (1:100) was purchased from Abcam, Cambridge, UK.

### Evaluation of Immunohistochemical Variables

For Immunohistochemistry staining, five fields of approximately 500 cells from each tumor were counted independently by 2 pathologists. BAP1 and DPP4 staining were reported separately according to the German semiquantitative scoring system. Briefly, depending on the percentage of staining intensity, the staining was classified into 4 groups: (no staining=0; weak staining = 1; moderate staining = 2; and strong staining = 3) and the percentage of stained cells (0% = 0; 1%-25% = 1; 26% 50% = 2; 51%-75% =3; and 76%-100% = 4). Final immunoreactive scores were determined by the formula: overall scores = intensity score × percentage score. The overall score ≤ 3 was defined as negative, >3 as positive.

PD-L1 expression was assessed in HCC cells as previously described 13. For neoplastic cells, the percentage of cells displaying unequivocal membranous staining was recorded, and tumors with >1% of positive cells were classified as positive.

### Differential genes expression and functional enrichment analysis

Limma package (version: 3.40.2) of R software was used to study the differential expression of mRNAs. The adjusted P-value was analyzed to correct for false positive results in TCGA. “Adjusted P < 0.05 and Log (Fold Change) >1 or Log (Fold Change)<-1” were defined as the thresholds for the screening of differential expression of mRNAs.

To further confirm the underlying function of potential targets, the data were analyzed by functional enrichment. Gene Ontology (GO) is a widely-used tool for annotating genes with functions. Kyoto Encyclopedia of Genes and Genomes (KEGG) Enrichment Analysis is a practical resource for analytical study of gene functions and associated high-level genome functional information. To better understand the carcinogenesis of mRNA, ClusterProfiler package (version: 3.18.0) in R was employed to analyze the GO function of potential targets and enrich the KEGG pathway.

### Follow-Up

Patients were followed regularly in the outpatient clinic and were monitored prospectively for recurrence according to a standard protocol as previously described 14. All patients were monitored prospectively by serum AFP, liver function, ultrasonography and chest X-ray every two months, and contrast enhanced computed tomography (CT) was performed every 6 months. Bone scan or magnetic resonance imaging (MRI) was performed if localized bone pain was reported. A diagnosis of recurrence was based on typical imaging appearance in CT and/or MRI scan and an elevated AFP level.

### Statistical analyses

The chi-square test or the Fisher exact probability test was used to evaluate categoric variables, and the Student t test was used to evaluate continuous variables. The cumulative overall survival (OS) rate was calculated using the Kaplan-Meier method and was compared using the log-rank test. Overall survival was calculated from the date of resection to the date of death regardless of the cause of death. Recurrence free survival (RFS) rate was calculated from the date of resection to the date when tumor recurrence was diagnosed or from date of the resection to the last visit, if recurrence was not diagnosed, and the patients were censored at the date of death or the date of last follow-up 14.

Statistical analyses were performed using the SPSS statistical software package (version 13.0; SPSS Inc., Chicago, IL). Two-tailed p values <0.05 were considered statistically significant.

## Results

### BAP1 somatic mutation and expression in HCC

**Figure 1:**
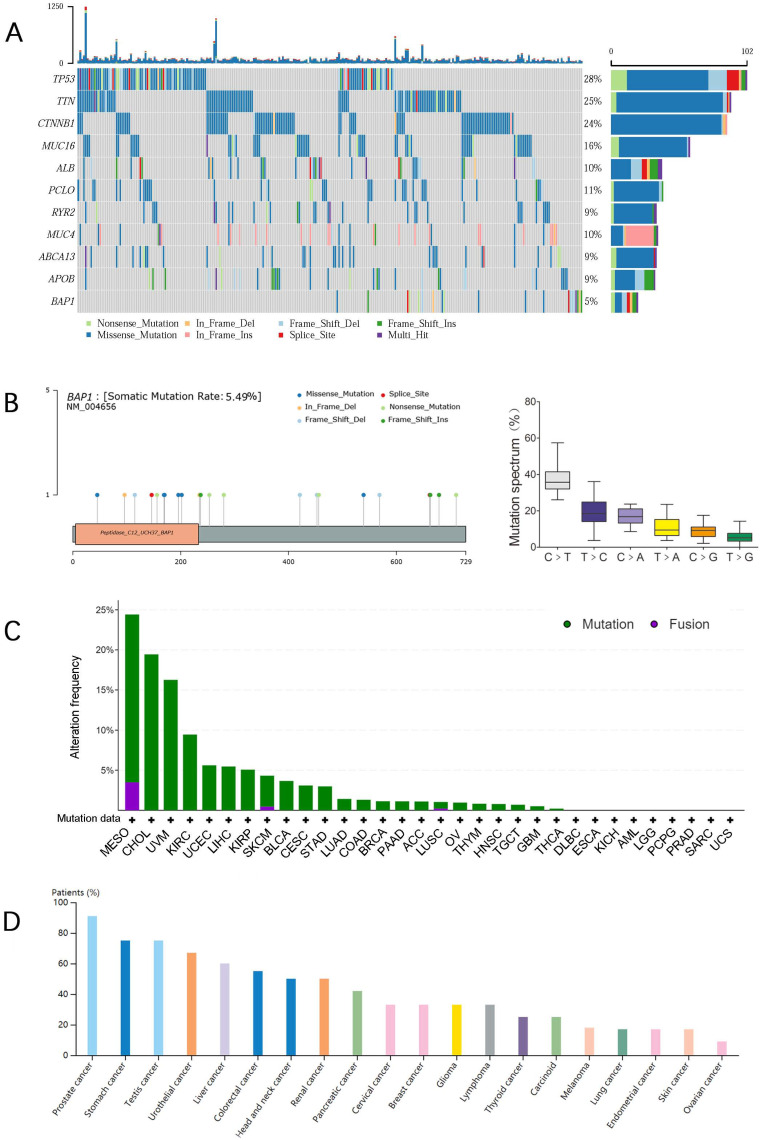
** A.** The oncoplot displaying of the somatic landscape of HCC cohort. **B.** Distribution of BAP1 mutations across the different protein domains was demonstrated, and C>T substitutions were the most predominant substitution type. **C.** The mutation frequency of BAP1 mutation in different cancer types. **D.** The frequency of expression trends of BAP1 across different cancers from Human Protein Atlas Dataset.

We performed an in silico analysis by reproducing TCGA database using the online cBioPortal platform (http://www.cbioportal.org/) as previously reported 15. After excluding cases without gene expression data (generated by RNA sequencing and shown in pre-normalized Z-scores) and somatic BAP1 mutation data (generated using genome sequencing), 352 HCC cases were entered in our analysis, including 20 cases with BAP1 mutations and 332 cases with wild-type BAP1. For both groups, survivals were plotted using Kaplan-Meier method. Figure 1A is oncoplot displaying of the somatic landscape of HCC cohort. Genes are ordered by their mutation frequency. Side bar plot shows log10 transformed Q-values estimated by MutSigCV. Mutation information of each gene in each sample was shown in the waterfall plot, where different colors with specific annotations at the bottom meant the various mutation types. The barplot above the legend exhibited the number of mutation burden.

A mutational spectrum analysis for mutations on exons in BAP1 genes was performed on the 20 mutated HCC. Distribution of BAP1 mutations across the different protein domains was demonstrated in Figure 1B, and the height of the blue bars correlates with the frequency of the observed mutation. We identified splicing (n=2), nonsense (n=5), missense (n=6), and frameshift (n=8) mutations in BAP1 mutated samples (Table 1). The allele frequency ranged from 14% to 82%. Within six different substitutions, C>T substitutions were the most predominant substitution type and T>G substitutions were the least substitution type in HCC with BAP1 mutation (Figure 1B).

**Figure 2:**
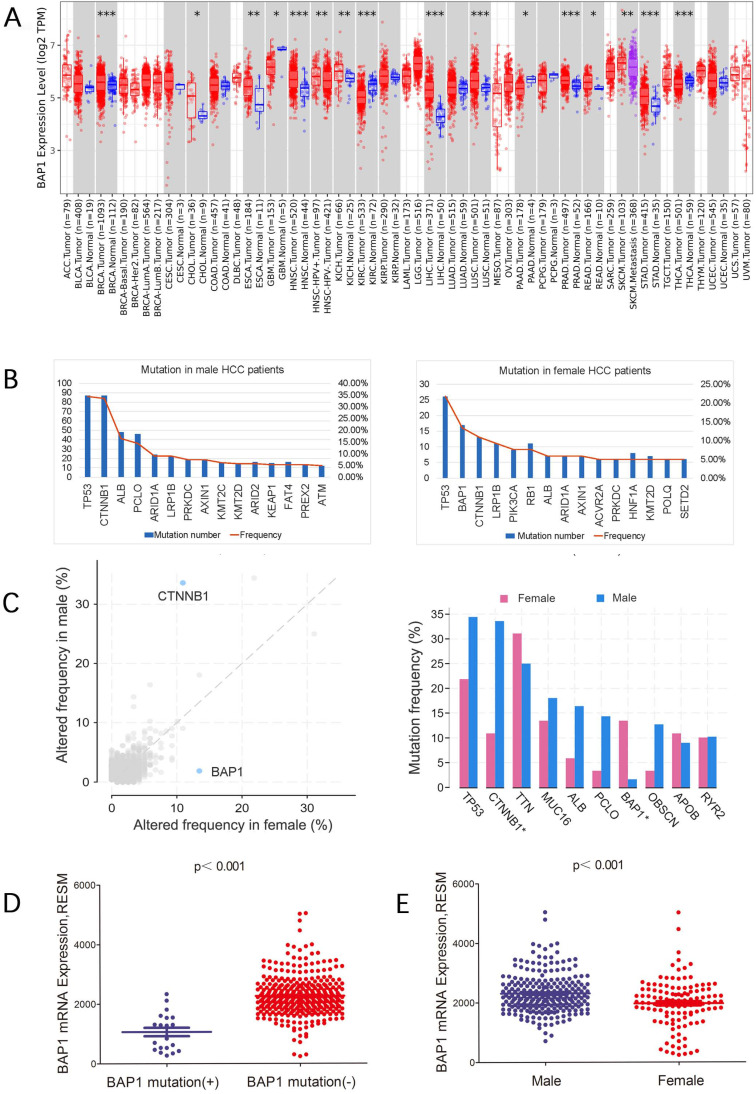
** A.** The differential expression of mRNA in cancer tissues and corresponding normal tissues in different types of cancer in the TCGA database, and the BAP1 mRNA expression was significantly higher in HCC tissues than in normal liver tissues. **B, C.** Different gene mutations were detected in male and female HCC patients. Comparison of gene mutation frequency between male and female patients revealed that besides TP53, CTNNB1 was most frequently mutated in male HCC patients while BAP1 was most frequently mutated in female patients. **D.** The mRNA expression of BAP1 was significantly lower in mutated HCCs than those without mutation. **E.** BAP1 mRNA expression was significantly lower in female group than in male patients.

Figure 1C showed the mutation frequency of BAP1 mutation in different cancer types. The mutation rate of BAP1 in HCC was 5.49%, higher than most of other cancers. In addition, the frequency of expression trends of BAP1 across different cancers from Human Protein Atlas Dataset was demonstrated in Figure 1D, and BAP1 expression was detected in more than 60% of HCC patients. Though BAP1 mutation was detected in most types of cancer tissue, however, the differential expression of mRNA in cancer tissues and corresponding normal tissues was not consistent in different types of cancer in the TCGA database (Figure 2A), and the BAP1 mRNA expression was significantly higher in HCC tissues than in normal liver tissues (Figure 2A).

### BAP1 somatic mutation and expression in male and female HCC patients

Different gene mutations were detected in male and female HCC patients. Comparison of gene mutation frequency between male and female patients revealed that besides TP53, CTNNB1 was most frequently mutated in male HCC patients while BAP1 was most frequently mutated in female patients (Figure 2B, C).

We further sought to confirm expression level of mutated genes in HCC. We found that the mRNA expression of BAP1 was significantly lower in mutated patients than those without mutation (Figure 2D). In addition, BAP1 mRNA expression was significantly lower in female group which had most frequently mutated BAP1 than in male patients (Figure 2E).

In our series of samples, BAP1 mutations were observed predominately in female patients (Table 2, p<0.001), and mutation rate of BAP1 was significantly high in white patients than in Asian patients (Table 2, p=0.040). There were no significant differences regarding age, TNM stage and histologic grade between patients with and without BAP1 mutation (Table 2).

### Expression of BAP1 is predictive of worse prognosis in HCC patients

**Figure 3:**
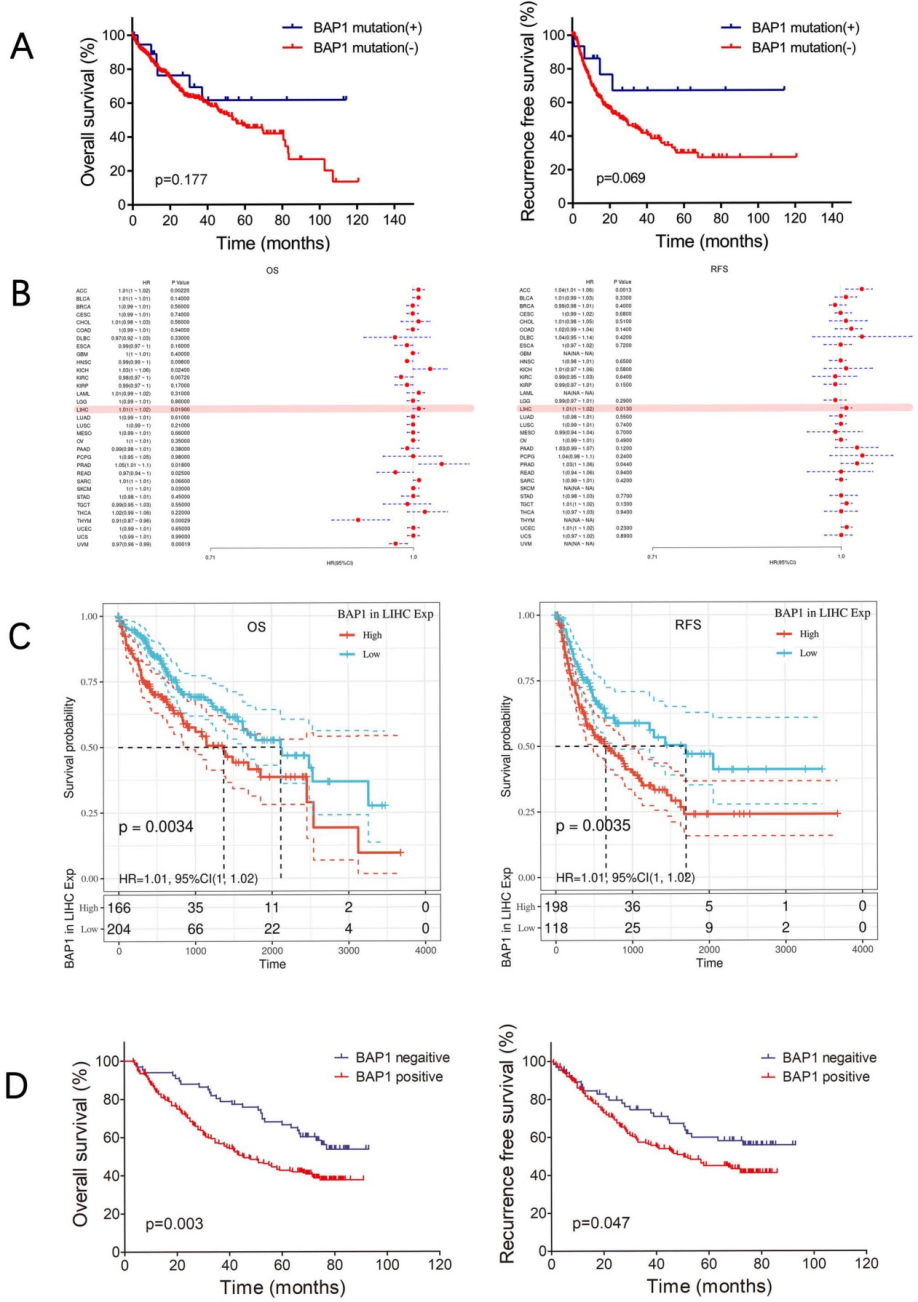
** A.** The OS and RFS of BAP1 mutated HCC tended to be higher than those of non-mutated patients. **B.** Data from TCGA database revealed negative correlation of BAP1 mRNA expression with the survival of HCC patient. **C.** Both the OS and RFS of HCC patients with low BAP1 mRNA expression was significantly higher than those of patients with high BAP1 expression. **D.** Kaplan-Meier survival analysis revealed that positive expression of BAP1 is associated with worse OS and RFS than patients with negative BAP1 expression.

The OS and RFS of BAP1 mutated HCC tended to be higher than those of non-mutated patients, though the differences were not significant, possibly due to limited number of mutated patients (Figure 3A). However, data from TCGA database revealed negative correlation of BAP1 mRNA expression with the survival of HCC patient (Figure 3B). Both the OS and RFS of HCC patients with low BAP1 expression was significantly higher than those of patients with high BAP1 expression (Figure 3C).

To further investigate whether BAP1 expression is relevant to human HCC prognosis, we examined the expression of BAP1 in 297 human HCC samples by using human tissue microarray. Among the 297 HCC samples, 231 (77.8%) samples showed positive staining for BAP1, while the other 66 samples showed negative staining for BAP1. Kaplan-Meier survival analysis revealed that positive expression of BAP1 is associated with worse OS and RFS than patients with negative BAP1 expression. The 1-, 3-, and 5-year OS rates after curative resection for BAP1 positive patients were 85.3%, 56.7% and 42.7%, significantly worse than those of BAP1 negative patients (93.9%, 78.8% and 66.5%, p=0.003, Figure 3D). The 1-, 3-, and 5-year RFS rates of BAP1 positive patients were also significantly worse than those of BAP1 negative patients (83.6%, 56.9% and 44.9% vs. 86.0%, 74.5% and 60.0%, p=0.047, Figure 3D).

In our study, multivariate analysis (Table 3) revealed that reduced BAP1 expression was an independent risk factor for better OS (p=0.006, HR: 1.78, 95% CI: 1.18-2.68) after curative resection, but not for RFS.

### Differential expressed genes in HCC with BAP1 mutation

**Figure 4:**
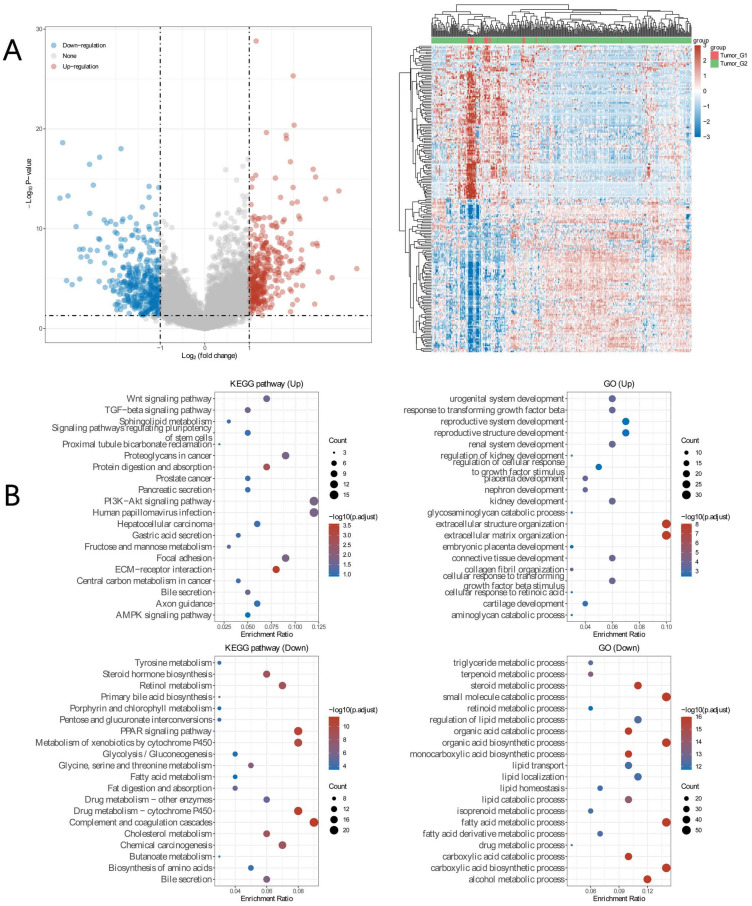
** A.** Transcriptional microarray analysis of 359 HCC cases from TCGA cohort was performed to explore DEGs profiles with BAP1 mutation. Based on BAP1 mutation, 345 genes were up-regulated and 361 genes down-regulated in the BAP1 mutation group after propensity analysis using limma package algorithm. **B.** Functional enrichment analysis including GO and KEGG pathways, was performed in DEGs.

To explore differential expressed genes (DEGs) profiles with BAP1 mutation, we performed transcriptional microarray analysis of 359 HCC cases from TCGA cohort. Volcano plots were constructed using fold-change values and adjusted P. The red point in the plot represents the over-expressed mRNAs and the blue point indicates the down-expressed mRNAs with statistical significance. Based on BAP1 mutation, 345 genes were up-regulated and 361 genes down-regulated in the BAP1 mutation group after propensity analysis using limma package algorithm (Figure 4A).

In addition, functional enrichment analysis including GO and KEGG pathways, was performed in DEGs (Figure 4B). GO analyses of DEGs revealed the biological processes most associated with small molecule catabolic process, fatty acid catabolic process, carboxylic acid biosynthetic process, steroid metabolic progress. KEGG function analysis of these genes revealed that they were mainly enriched in the drug metabolism, metabolism of xenobiotics cytochrome 450, cholesterol metabolism, steroid hormone biosynthesis, central carbon metabolism in cancer, PI3K-Akt signaling pathway, and PPAR signaling pathway, which are usually associated with ferroptosis 16-18.

### BAP1 mutation and ferroptosis

**Figure 5:**
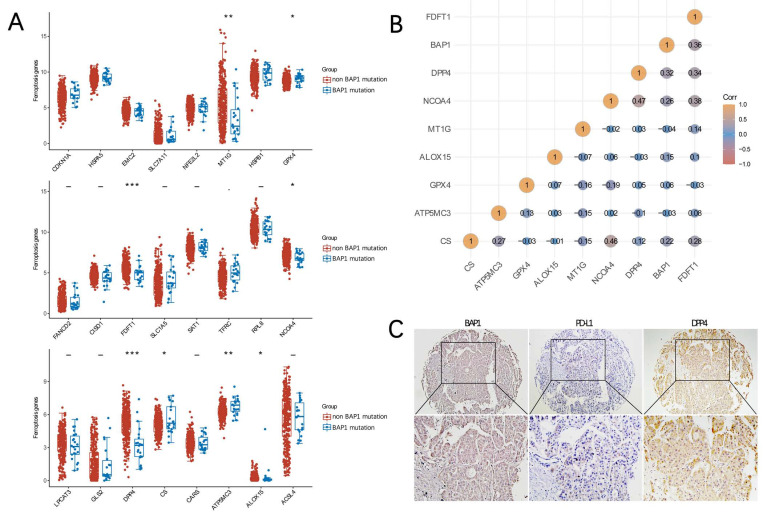
** A.** The mRNA expressions of FRGs in HCC with and without BAP1 mutation were demonstrated. **B.** Spearman's correlation analysis of the correlation between BAP1 gene expression and FRGs expression. **C.** The expression of BAP1, DPP4 and PD-L1 in HCC tissues were assessed by immunohistochemistry staining of human HCC tissue microarray.

Ferroptosis related genes (FRGs) are derived from Ze-Xian Liu et al.'s systematic analysis of the aberrances and functional implications of Ferroptosis in Cancer 19. The mRNA expressions of FRGs in HCC with and without BAP1 mutation were demonstrated in Figure 5A. The expression of GXP4 and CS was significantly higher in BAP1 mutated HCC than in non-mutated HCC, while the expression of ALOX15, FDFT1, NCOA4, DPP4 was significantly lower in BAP1 mutated HCC than in non-mutated HCC, indicating that ferroptosis was promoted in non-mutated HCC (*p<0.05, **p<0.01, ***p<0.001).

We further used Spearman's correlation analysis to describe the correlation between BAP1 gene expression and FRGs expression. Spearman correlation analysis revealed that BAP1 gene expression was positively correlated with the expression of DPP4 and FDFT1 (Figure 5B). To further analyze the relationship, the expression of BAP1 and DPP4 in HCC tissues were assessed by immunohistochemistry staining of human HCC tissue microarray (Figure 5C), and a positive correlation between them was confirmed.

### BAP1 expression correlates with immune status in HCC

**Figure 6:**
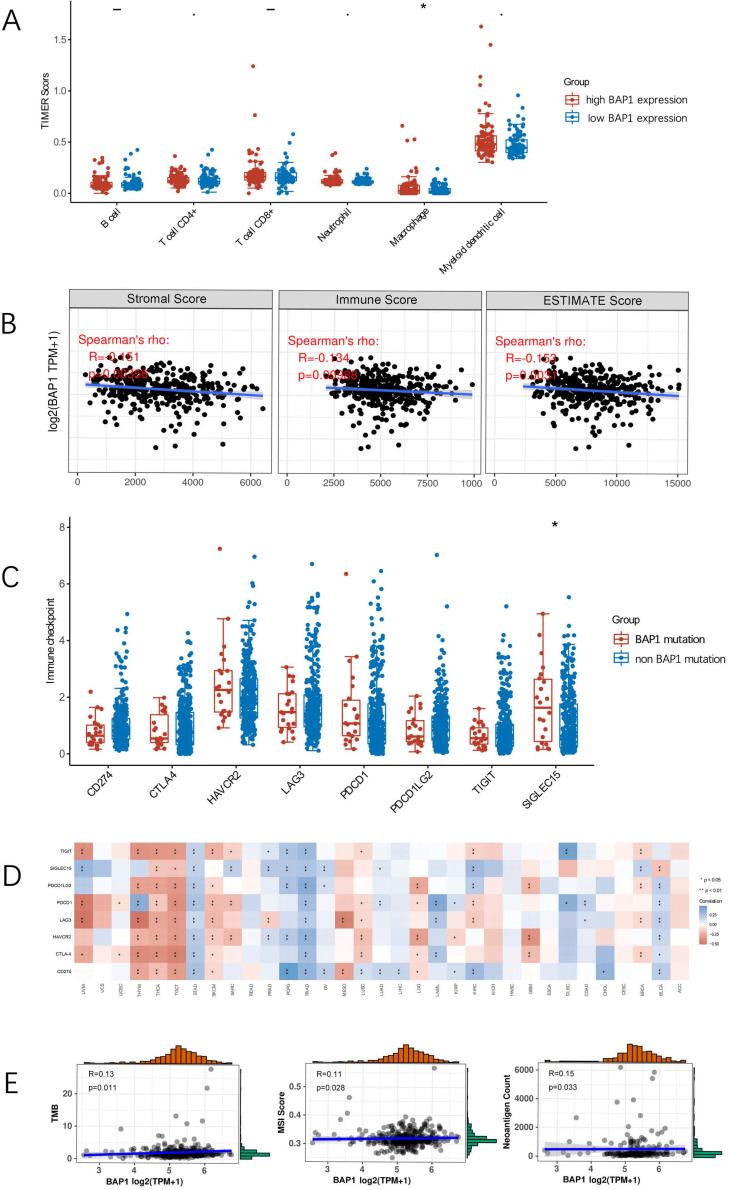
** A.** HCC with high BAP1 expression was significantly associated with the infiltration of macrophage. **B.** The mRNA expression of BAP1 was negatively associated with ImmuneScore, StromalScore and ESTIMATEScore. **C.** The comparison of the expression of immune checkpoint molecules in HCC with or without BAP1 mutation. **D.** A heat map of the correlation between mRNA level of BAP1 and multiple immune checkpoint genes in different types of cancer. The expression of BAP1 mRNA was only significantly correlated with the expression of PD-L1 in HCC. **E.** Correlation analysis of BAP1 mRNA expression and level of TMB, MSI and NC.

Tumor-infiltrating lymphocytes (TILs) are independent predictors of survival in cancers. It is unclear whether mutation of BAP1 can lead to the recruitment of more immune cells into the tumor microenvironment and thus affect the prognosis of HCC. We explored the associations between BAP1 expression and TILs by TIMER analysis. As depicted in Figure 6A, HCC with high BAP1 expression was significantly associated with the infiltration of macrophage.

By using the ESTIMATE algorithm, we assessed stromal and immune scores, which are represented for the immune or stromal components in tumor microenvironment (TME). As shown in Figure 6B, the mRNA expression of BAP1 was negatively associated with ImmuneScore (R=-0.134, p=0.009), StromalScore (R=-0.151, p=0.004), and ESTIMATEScore (R=-0.153, p=0.003), indicating that high BAP1 expression reflected an immunosuppressive state in HCC.

### Correlation of BAP1 mutation and expression with immune checkpoint molecules

The comparison of the expression of immune checkpoint molecules in HCC with or without BAP1 mutation revealed that there were no significant differences in most of immune checkpoint molecules (Figure 6C), including CD274 (PD-L1) (p=0.10), probably due to the limited number of mutated cases. Figure 6D demonstrated a heat map of the correlation between mRNA level of BAP1 and multiple immune checkpoint genes in different types of cancer. The expression of BAP1 mRNA was only significantly correlated with the expression of PD-L1 in HCC.

Since PD-L1 expression on tumor cells has been used widely as a predictive marker for efficacy of ICI therapy, to further analyze the relationship, the expression of BAP1 and PD-L1 in HCC tissues were assessed by immunohistochemistry staining of human HCC tissue microarray (Figure 5C). Positive staining of PD-L1 was detected in 23.4% (93 of 297) of HCC samples. The expression of PD-L1 was significantly higher in BAP1 positive patients than in BAP1 negative patients (80/151 vs 13/53, p=0.024).

Correlation analysis of BAP1 mRNA expression and tumor mutational burden (TMB), microsatellite instability (MSI), neoantigen count (NC), was presented in Figure 6E. BAP1 expression was weak correlated with TMB, MSI and NC. Since BAP1 expression is positively correlated with the expression of PD-L1, as well as TMB/MSI/NC, BAP1 may be a useful indicator for HCC patients to receive ICI treatment.

### Comparison of clinicopathologic characteristics of BAP1 positive and negative patients

The clinicopathologic characteristics of BAP1 positive and negative patients were compared, and Table 4 showed the baseline demographic data and tumor characteristics of them. There were no significant differences between these two groups regarding gender, age, HBsAg status, APF level, GGT and ALT level, cirrhosis, tumor capsule, tumor differentiation, vascular invasion, and tumor size. HCC patients with positive BAP1 expression tends to have multiple tumors than patients with negative BAP1 (p=0.017).

## Discussion

BAP1 is a multifunctional tumor suppressor involved in chromatin remodeling, DNA damage repair, cell cycle control, regulated cell death, and the immune response 20. Given the widespread functional role of BAP1 in cellular pathways implicated in cancer, it is not surprising that the BAP1 gene is altered in a variety of tumors. Germline mutations are either inherited or de novo mutations. Numerous studies have now confirmed and expanded on the direct link of BAP1 germline mutations to a hereditary cancer syndrome characterized by a predisposition to mesothelioma, uveal melanoma, and less frequently cutaneous melanoma, as well as clear cell renal cell carcinoma 21, which are the core cancer types in the BAP1 cancer syndrome. However, germline BAP1 mutations are extremely rare in HCC and are found in only about 0.68% of HCC cases 7. In contrast, somatic BAP1 mutations are frequent in various human cancers, including uveal melanoma and mesothelioma, and have been noted in breast, lung, and renal cancers. Though somatic BAP1 mutation have not previously been appreciated as a significant alteration in HCC, in fact, somatic BAP1 mutations are more frequent than germline mutations and are found in about 6% of HCC cases.

Somatic BAP1 mutations in HCC were present predominantly in women, and mutation rate of BAP1 was significantly high in white patients than in Asian patients, whether the sex and race bias in BAP1 mutation status is the result of a biological difference or a sampling issue remains unclear, but a meta-analysis including 12 studies with 3447 participants also revealed that BAP1 mutation was more common in women than in men in different types of cancer 22. Though somatic BAP1 mutations in uveal melanoma and mesothelioma were associated with worse OS and higher metastatic risk in comparison to BAP1 wild-type tumor, however, in this study, somatic BAP1 mutations interestingly tended to be associated with better OS and DFS in comparison to BAP1 wild-type HCC. Further analysis revealed that somatic BAP1 mutations in HCC resulted in loss of BAP1 expression, and the OS and RFS of patients with high BAP1 expression was significantly lower than that of patients with low BAP1 expression.

We further examined the expression of BAP1 in 297 human HCC samples by using human tissue microarray. HCC patients with positive BAP1 expression tended to have multiple tumors than patients with negative BAP1, despite that, there were no significant differences between these two groups regarding gender, age, and other clinicopathologic characteristics. Kaplan-Meier survival analysis also revealed a positive correlation of BAP1 expression with worse OS and RFS, and multivariate analysis revealed that reduced BAP1 expression was an independent risk factor for better OS. All these findings are contradictory to other types of cancer, and indicating that BAP1 may play different roles in development of HCC.

The biological consequences of BAP1 levels in the development of HCC are still unclear. BAP1 promotes double-strand DNA repair by homologous recombination, a key process to reduce genetic damage and prevent cancer. In the nucleus, BAP1 binds to specific proteins, which are assembled in multiprotein complexes that in turn may associate with other tissue-specific transcriptional regulators and modulates several cellular activities 7. Although physiologic BAP1 levels are required for cells to execute apoptosis, however, BAP1 was recently shown to have an anti-apoptotic role in the liver 23, which could explain our observation of an increased expression of BAP1 in HCC compared to normal liver. In addition, BAP1 may also inhibit cell death induced by unresolved metabolic stress. This prosurvival role of BAP1 depends on its de-ubiquitinating activity and correlates with its ability to dampen the metabolic stress-induced unfolded protein response (UPR) transcriptional network 24. In Dai's study, the authors found that the loss of function of BAP1 had a proapoptotic effect in mouse embryonic stem cells, fibroblasts, liver, and pancreas, but not in melanocytes and mesothelial cells 24. These findings suggest a complex role for BAP1 in cancer that is context- and lineage-dependent and may differ among cancer types and species 7.

Recent studies have demonstrated that BAP1 is also involved in several aspects of cellular metabolism, including ferroptosis 26, a recently identified form of regulated cell death that is induced by metabolic stress from cystine reduction and increased reactive oxygen species 27-29. Epidemiological evidence suggests that high dietary iron intake increases the risk of several cancer types including HCC 30, and ferroptosis has been implicated in the development and therapeutic responses of various types of tumors 31. The extent to which ferroptosis affects tumor biology is unclear, and ferroptosis has a dual role in tumorigenesis, which depends on the release of damage-associated molecular patterns and the activation of immune response triggered by ferroptotic damage within the tumor microenvironment 32. Several studies have found correlations between ferroptosis and mutations in cancer-relevant genes, including TP53. In this study, BAP1 mutated HCC showed reduced ability to promote ferroptosis and high BAP1 expression is correlated with ferroptosis. Since iron-rich tumors such as HCC might be particularly responsive to agents that promote ferroptosis, BAP1 may be characterized as biomarkers of response to ferroptosis-promoting therapy, although their clinical significance remains unknown 30.

Recently, with the rapid development of cancer immunotherapy, several studies have investigated the association between BAP1 alterations and immunological phenotypes 20, 33. Loss of BAP1 is known to alter chromatin architecture exposing the DNA to damage and also impairing the DNA repair machinery, which will drive genomic instability and dysregulate tumor microenvironment, leads to the increased secretion of cytokines, including interferons that promote tumor-antigen presentation and trigger recruitment of T lymphocytes to destroy tumor cells 33. As a response, tumor cells evade this immune surveillance by increased expression of immune checkpoint receptors. Therefore, loss of BAP1 expression is associated with an immunosuppressive microenvironment in uveal melanoma, with implications for immunotherapy development 33-35.

In contrast, BAP1 expression in HCC was associated with significantly increased infiltration of macrophage, and BAP1 expression was negatively correlated with ESTIMATEScore. It's possible that ferroptotic damage can trigger inflammation-associated immunosuppression in the tumor microenvironment 32, thus favoring HCC growth and influencing the survival. In addition, BAP1 expression was positively correlated with elevated levels of TMB, MSI and NC, as well as PD-L1 in HCC, the most extensively studied predictive biomarkers for efficacy of ICI therapy 36-40. Although the particular role of BAP1 in immune regulation remains unspecified in HCC, BAP1 is expressed and functional across many cell-types and tissues, including those of the immune system 41. BAP1 may be a beneficial complement to immune checkpoints for predicting ICI therapy efficacy in HCC.

In summary, our study revealed that BAP1 expression was a prognostic indicator for worse survival of HCC patients, and may be characterized as a biomarker for ICI therapies. In addition, BAP1 expression may also be used to evaluate response to ferroptosis-promoting therapy, but future studies are needed to clarify the role and mechanism of BAP1 in immune and ferroptosis regulation.

## Figures and Tables

**Table 1:** Overview of BAP1 Mutations Detected in 20 HCC samples

Sample	Somatic	Protein change	Mutation type	copy	Cosmic occurrence	Allele frequency
1	Yes	G45R	Missense	Diploid	1	0.26
	Yes	K711*	Nonsense	Diploid	1	0.23
2	Yes	M115Rfs*9	FS del	Shallow deletion		0.43
3	Yes	X146_splice	Splice	Shallow deletion		0.62
4	Yes	Q156*	Nonsense	Diploid	1	0.37
	Yes	Q456*	Nonsense	Diploid		0.46
5	Yes	H169Q	Missense	Shallow deletion	5	0.59
6	Yes	F170V	Missense	Diploid	4	0.14
7	Yes	W196G	Missense	Shallow deletion	1	0.57
8	Yes	W202L	Missense	Shallow deletion		0.64
9	Yes	P235_R237del	IF del	Shallow deletion		0.82
10	Yes	Q253*	Nonsense	Shallow deletion	2	0.58
11	Yes	L259Rfs*2	FS del	Deep deletion		0.46
12	Yes	Q280*	Nonsense	Shallow deletion	1	0.64
13	Yes	A359Tfs*68	FS del	Shallow deletion		0.31
14	Yes	K421Rfs*9	FS del	Shallow deletion	1	0.35
15	Yes	K453Rfs*15	FS del	Diploid	2	0.32
16	Yes	L539Q	Missense	Diploid		0.16
17	Yes	V569Cfs*2	FS del	Shallow deletion		0.35
18	Yes	X662_splice	Splice	Shallow deletion		0.30
19	Yes	D663*	FS ins	Shallow deletion		0.45
20	Yes	I679Yfs*38	FS ins	Shallow deletion		0.34

**Table 2:** Comparison of patient demographics and clinical characteristics between HCC patients with or without BAP1 somatic mutation

Variable	Number of patients (%)		*p*
	BAP1 mutation (+) (n=20)	BAP1 mutation (-) (n=332)	
**Gender (%)**			<0.001
male	4 (20.0)	233 (70.2)	
female	16(80.0)	99 (29.8)	
**Age, yrs (%)**			0.486
≤50	6 (30.0)	77 (23.2)	
>50	14 (70.0)	255 (77.8)	
**TNM stage (%)**			1.000
I-II	13 (76.5)	240(73.6)	
III-IV	4 (23.5)	86 (26.4)	
**Race (%)**			0.040
White	15 (75.0)	157 (51.3)	
Asian	5 (25.0)	149(48.7)	
**Histologic Grade (%)**			0.484
I-II	11 (55.0)	206 (62.8)	
III-IV	9 (45.0)	122 (37.2)	

**Table 3:** Comparison of patient demographics and clinical characteristics between BAP1 positive and negative patients

Variable	Number of patients (%)		*p*
	BAP1 positive (n=231)	BAP1 negative (n=66)	
**Gender (%)**			0.335
male	203 (94)	55 (86)	
female	28 (6)	11 (14)	
**Age, yrs (%)**			0.852
≤50	109 (70)	32 (49)	
>50	122 (30)	34 (51)	
**HBsAg (%)**			0.105
positive	189 (72)	48 (81)	
negative	42 (28)	18 (19)	
**AFP, ng/mL (%)**			0.922
≤20	79 (49)	23 (32)	
>20	152 (51)	43 ( 68)	
**ALT, U/L (%)**			0.733
≤75	213 (89)	60 (92)	
>75	18 (11)	6 (1)	
**GGT, U/L (%)**			0.924
≤50	93 (36)	27 (41)	
>50	138 (64)	39 (59)	
**Cirrhosis (%)**			0.278
yes	192 (81)	51 (82)	
no	39 (19)	15 (18)	
**Tumor size, cm (%)**			0.491
≤5	129 (51)	40 (58)	
>5	102 (49)	26 (42)	
**Tumor number (%)**			0.017
single	175 (76)	59 (89)	
multiple	56 (24)	7 (11)	
**Tumor capsule (%)**			0.592
yes	93 (64)	29 (58)	
no	138 (36)	37 (42)	
**Vascular invasion (%)**			0.171
yes	61 (17)	12 (26)	
no	170 (83)	54 (74)	
**Tumor differentiation (%)**			0.973
I-II	157(81)	45 (66)	
III-IV	74 (19)	21 (34)	

ALT: alanine aminotransferase; GGT: γ-glutamyltransferase; AFP: a-fetoprotein.

**Table 4:** Multivariate analysis of risk factors related to OS of HCC patients

Variable	HR	95% CI	*p*
**AFP, ng/mL**			
≤20	1		
>20	1.57	1.10-2.23	0.013
**Cirrhosis**			
no	1		
yes	1.62	1.03-2.54	0.038
**Tumor size, cm**			
≤5	1		
>5	1.95	1.43-2.67	<0.001
**Vascular invasion**			
no	1		
yes	1.79	1.27-2.52	0.001
**BAP1 expression**			
Negative	1		
Positive	1.78	1.18-2.68	0.006
**Tumor differentiation**			
I-II	1		
III-VI	1.40	1.02-1.94	0.039

## Abbreviations

BAP1: BRCA1-Associated Protein 1
HCC: hepatocellular carcinoma
AFP: a-fetoprotein
ICI: immune checkpoint inhibitors
RNA-seq: RNA-sequencing
TCGA: The Cancer Genome Atlas
ESTIMATE: Estimation of STromal and Immune cells in Malignant Tumor tissues using Expression data
CT: computed tomography
MRI: magnetic resonance imaging
PD-L1: programmed death-ligand1
GO: Gene Ontology
KEGG: Kyoto Encyclopedia of Genes and Genomes
DEGs: differential expressed genes
TMB: tumor mutation burden
MSI: microsatellite instability
NC: neoantigen count
FRGs: Ferroptosis related genes
TILs: tumor infiltrating lymphocytes
TME: tumor microenvironment
OS: overall survival
DFS: disease-free survival
ALT: alanine aminotransferase
GGT: γ-glutamyl transpeptidase

## References

[2] Riordan JD, Feddersen CR, Tschida BR (2018). Chronic liver injury alters driver mutation profiles in hepatocellular carcinoma in mice. Hepatology.

[3] Liu KX, Hong JG, Wu R (2021). Clinical Benefit of Antiviral Agents for Hepatocellular Carcinoma Patients With Low Preoperative HBV-DNA Loads Undergoing Curative Resection: A Meta-Analysis. Front Oncol.

[4] Carbone M, Yang H, Pass HI (2013). BAP1 and cancer. Nat Rev Cancer.

[5] Jensen DE, Proctor M, Marquis ST (1998). BAP1: a novel ubiquitin hydrolase which binds to the BRCA1 RING finger and enhances BRCA1-mediated cell growth suppression. Oncogene.

[6] Brekken RA (2020). Loss of BAP1 Leads to More YAPing in Pancreatic Cancer. Cancer Res.

[7] Carbone M, Harbour JW, Brugarolas J (2020). Biological Mechanisms and Clinical Significance of BAP1 Mutations in Human Cancer. Cancer Discov.

[8] He M, Chaurushiya MS, Webster JD (2019). Intrinsic apoptosis shapes the tumor spectrum linked to inactivation of the deubiquitinase BAP1. Science.

[9] Bi KW, Wei XG, Qin XX (2020). BTK Has Potential to Be a Prognostic Factor for Lung Adenocarcinoma and an Indicator for Tumor Microenvironment Remodeling: A Study Based on TCGA Data Mining. Front Oncol.

[10] Dong ZR, Chen ZQ, Yang XY (2021). RECK expression is associated with angiogenesis and immunogenic Tumor Microenvironment in Hepatocellular Carcinoma, and is a prognostic factor for better survival. J Cancer.

[11] Li T, Qin LX, Gong X (2013). Hepatitis B virus surface antigen-negative and hepatitis C virus antibody-negative hepatocellular carcinoma: clinical characteristics, outcome, and risk factors for early and late intrahepatic recurrence after resection. Cancer.

[12] Wang CH, Guo ZY, Chen ZT (2015). TMPRSS4 facilitates epithelial-mesenchymal transition of hepatocellular carcinoma and is a predictive marker for poor prognosis of patients after curative resection. Sci Rep.

[13] Calderaro J, Rousseau B, Amaddeo G (2016). Programmed death ligand 1 expression in hepatocellular carcinoma: Relationship With clinical and pathological features. Hepatology.

[14] Li T, Wang SK, Zhou J (2016). Positive HBcAb is associated with higher risk of early recurrence and poorer survival after curative resection of HBV-related HCC. Liver Int.

[15] Sun C, Zhao C, Li S (2018). EZH2 Expression is increased in BAP1-mutant renal clear cell carcinoma and is related to poor prognosis. J Cancer.

[16] Wu ZH, Tang Y, Yu H (2021). The role of ferroptosis in breast cancer patients: a comprehensive analysis. Cell Death Discov.

[17] Venkatesh D, O'Brien NA, Zandkarimi F (2020). MDM2 and MDMX promote ferroptosis by PPARα-mediated lipid remodeling. Genes Dev.

[18] Yi J, Zhu J, Wu J (2020). Oncogenic activation of PI3K-AKT-mTOR signaling suppresses ferroptosis via SREBP-mediated lipogenesis. Proc Natl Acad Sci U S A.

[19] Liu Z, Zhao Q, Zuo ZX (2020). Systematic Analysis of the Aberrances and Functional Implications of Ferroptosis in Cancer. iScience.

[20] Louie BH, Kurzrock R (2020). BAP1: Not just a BRCA1-associated protein. Cancer Treat Rev.

[21] Feng Z, Zhang L, Qi Z (2020). Identifying BAP1 Mutations in Clear-Cell Renal Cell Carcinoma by CT Radiomics: Preliminary Findings. Front Oncol.

[22] Luchini C, Veronese N, Yachida S (2016). Different prognostic roles of tumor suppressor gene BAP1 in cancer: A systematic review with meta-analysis. Genes Chromosomes Cancer.

[23] Artegiani B, van Voorthuijsen L, Lindeboom R (2019). Probing the Tumor Suppressor Function of BAP1 in CRISPR-Engineered Human Liver Organoids. Cell Stem Cell.

[24] Dai F, Lee H, Zhang Y (2017). BAP1 inhibits the ER stress gene regulatory network and modulates metabolic stress response. Proc Natl Acad Sci U S A.

[25] Schoumacher M, Le Corre S, Houy A (2016). Uveal melanoma cells are resistant to EZH2 inhibition regardless of BAP1 status. Nat Med.

[26] Han A, Purwin TJ, Aplin AE (2021). Roles of the BAP1 tumor suppressor in cell metabolism. Cancer Res.

[27] Zheng J, Conrad M (2020). The Metabolic Underpinnings of Ferroptosis. Cell Metab.

[28] Wu Y, Zhang S, Gong X (2020). The epigenetic regulators and metabolic changes in ferroptosis-associated cancer progression. Mol Cancer.

[29] Shi Z, Zhang L, Zheng J (2021). Ferroptosis: Biochemistry and Biology in Cancers. Front Oncol.

[30] Fonseca-Nunes A, Jakszyn P, Agudo A (2014). Iron and cancer risk-a systematic review and meta-analysis of the epidemiological evidence. Cancer Epidemiol Biomarkers Prev.

[31] Wu Y, Yu C, Luo M (2020). Ferroptosis in Cancer Treatment: Another Way to Rome. Front Oncol.

[32] Chen X, Kang R, Kroemer G (2021). Broadening horizons: the role of ferroptosis in cancer. Nat Rev Clin Oncol.

[33] Shrestha R, Nabavi N, Lin YY (2019). BAP1 haploinsufficiency predicts a distinct immunogenic class of malignant peritoneal mesothelioma. Genome Med.

[34] Figueiredo CR, Kalirai H, Sacco JJ (2020). Loss of BAP1 expression is associated with an immunosuppressive microenvironment in uveal melanoma, with implications for immunotherapy development. J Pathol.

[35] Ladanyi M, Sanchez Vega F, Zauderer M (2019). Loss of BAP1 as a candidate predictive biomarker for immunotherapy of mesothelioma. Genome Med.

[36] Wang Y, Zhao Z, Zhuang J (2021). Prognostic Value of Autophagy, Microsatellite Instability, and KRAS Mutations in Colorectal Cancer. J Cancer.

[37] Gao C, Li H, Liu C, Xu X (2021). Tumor Mutation Burden and Immune Invasion Characteristics in Triple Negative Breast Cancer: Genome High-Throughput Data Analysis. Front Immunol.

[38] Ma X, Zhang Y, Wang S (2021). Predictive value of tumor mutation burden (TMB) with targeted next-generation sequencing in immunocheckpoint inhibitors for non-small cell lung cancer (NSCLC). J Cancer.

[39] Wang K, Lu C, Sun JN (2021). Primary Hepatic Squamous Cell Carcinoma with High Microsatellite Instability Shows Good Response to Programmed Cell Death 1 Inhibitor as Adjuvant Therapy. Hepatology.

[40] Xue JS, Liu H, Meng GX (2021). Prognostic value of soluble programmed cell death-1 (sPD-1) and soluble programmed cell death ligand-1 (sPD-L1) for hepatocellular carcinoma: a systematic review and meta-analysis. Cancer Immunol Immunother.

[41] Lin YH, Liang Y, Wang H (2021). Regulation of B Lymphocyte Development by Histone H2A Deubiquitinase BAP1. Front Immunol.

